# Vibrotactile Sensory Substitution Elicits Feeling of Ownership of an Alien Hand

**DOI:** 10.1371/journal.pone.0050756

**Published:** 2012-11-30

**Authors:** Marco D’Alonzo, Christian Cipriani

**Affiliations:** The BioRobotics Institute, Scuola Superiore Sant’Anna, Pontedera, Italy; University of Bologna, Italy

## Abstract

Tactile feedback plays a key role in the attribution of a limb to the self and in the motor control of grasping and manipulation. However, due to technological limits, current prosthetic hands do not provide amputees with cutaneous touch feedback. Recent findings showed that amputees can be tricked into experiencing an alien rubber hand as part of their own body, by applying synchronous touches to the stump which is out of view, and to the rubber hand in full view. It was suggested that similar effects could be achieved by using a prosthesis with touch sensors that provides synchronous cutaneous feedback through an array of tactile stimulators on the stump. Such a prosthesis holds the potential to be easily incorporated within one’s body scheme, because it would reproduce the perceptual illusion in everyday usage. We propose to use sensory substitution – specifically vibrotactile – to address this issue, as current haptic technology is still too bulky and inefficient. In this basic study we addressed the fundamental question of whether visuo-tactile modality mismatch promotes self-attribution of a limb, and to what extent compared to a modality-matched paradigm, on normally-limbed subjects. We manipulated visuo-tactile stimulations, comprising combinations of modality matched, modality mismatched, synchronous and asynchronous stimulations, in a set of experiments fashioned after the Rubber Hand Illusion. Modality mismatched stimulation was provided using a keypad-controlled vibrotactile display. Results from three independent measures of embodiment (questionnaires, pointing tests and skin conductance responses) indicate that vibrotactile sensory substitution can be used to induce self-attribution of a rubber hand when synchronous but modality-conflicting visuo-tactile stimulation is delivered to the biological finger pads and to the equivalent rubber hand phalanges.

## Introduction

The sense of body ownership refers to the particular perceptual status that identifies part of the body as self. Such self-attribution is mediated by multi-sensory perceptual correlations [1]-[3]; e.g. the attribution of a visible hand to the self depends on a match between the afferent somatic signals and visual feedback from the hand. The Rubber Hand Illusion (RHI) is a perceptual illusion which elicits a feeling of ownership of an alien rubber hand; this effect was first shown by Botvinick and Cohen [1], and can be induced in an individual when a fake but realistic hand, placed in full view, is stroked while synchronously stroking the person’s own hand, which is hidden from view [1]. Specifically it was shown that after synchronous visuo-tactile stimulations, the perceived location of the participant’s hand shifted towards the rubber hand. This illusion does not occur when the rubber hand and the participant’s own hand are stroked asynchronously (i.e. when temporal delays are longer than 300 ms, as reported by [4]). Ehrsson and colleagues, measured the neural counterpart of this RHI by means of fMRI and demonstrated that the feeling of hand ownership is reflected in neural activity in the premotor cortex, hence suggesting that self-identification of the alien hand as a part of own body results from a multisensory integration in parieto–cerebellar regions and a recalibration of proprioceptive representations within the peripersonal space (i.e., the spatial area within reach of limbs of an individual) [5].

The RHI is a particularly interesting finding for prosthetics: in fact the restoration of motor and sensory functions of a lost arm with an artificial substitute that *is felt as* and acts like the biological limb represents one of the key goals in this field. A recent study by Ehrsson and colleagues demonstrated the possibility of eliciting the RHI in transradial amputees by stroking specific points on the residual limb [6]. The study suggests that similar effects can be achieved with a prosthesis with artificial sensors that provides synchronous and physiologically relevant coetaneous touch feedback through an array of tactile stimulators on the stump. The envisioned prosthesis, besides providing tactile feedback that could enhance volitional control [7], holds the potential to be easily incorporated within one’s body scheme because it would reproduce the perceptual illusion every time the prosthesis touches something, i.e. throughout everyday usage. The translation to the clinical practice of the non-invasive, perceptual-tricking approach proposed by Ehrsson is tied to non-trivial technological issues. In order to deliver physiologically relevant touch feedback, tactile stimulators capable of displaying stimuli in the same modality of the sensory events on the artificial fingers, i.e. *modality-matched* (e.g. pressure to pressure), would be necessary. This is not possible with current haptic robotic technology, wherein miniaturization, weight, and energy shortcomings hamper its exploitation in portable systems. Even the state of art multifunction haptic stimulator for upper limb prosthetics [8], would be excessively bulky to be applied to the forearm of transradial amputees in an array fashion.

We propose to use *sensory substitution* (or *modality mismatched* feedback) to tackle this problem: sensory substitution is a method to provide sensory information to the body through a sensory channel different from that normally used (e.g. substitute touch with hearing), or through the same channel but in a different modality e.g. substitute touch with vibration. If sensory substitution elicited the feeling of body ownership of a hand, we can expect that Ehrsson’s original idea could actually be translated into the clinic. Indeed miniature, inexpensive and reliable haptic arrays, like mobile-phone **vibrators**, could be easily fitted into a prosthesis equipped with tactile sensors [9], [10]. The scientific question arising from our proposal and which is still unanswered, is whether a visuo-tactile modality mismatch can still promote self-attribution of a limb and to which extent compared to a modality-matched paradigm. To answer this fundamental question in this work we performed experiments involving normally-limbed subjects. Previous studies have proven that the RHI may occur if using robot hands [11] or virtual arms [12] instead of human-like rubber hands and also when stimulating skin areas over surgically redirected sensory nerves through haptic stimulators [13] in targeted reinnervation amputees [14]. Armel and Ramachandran [15] argued that the illusion is the result of a purely bottom-up mechanism which associates synchronous visuo-tactile events; in other words any object could be self-attributed, simply due to the strong statistical correlations between different sensory modalities (vision and tactile). In contrast, other studies showed that top-down mechanisms and body representations are also involved: if the rubber hand is not placed in anatomically correct position [5], [16], [17], or if the hand is a non-corporeal object [18], or if the direction of the brushstrokes on the two hands is not the same [19], the illusion is inhibited. In all of these studies modality matched stimulation and/or spatially mismatched stimulation was delivered.

The study by Padilla et al. [20], although very preliminary, seemed to support the argument that RHI is possible with vibrotactile substitution, within active virtual reality sessions. In this work we employed the original RHI experiment by Botvinick and Coehn [1] and manipulated visuo-tactile stimulations, comprising of combinations of modality matched, modality mismatched, synchronous and asynchronous stimulations. As described in previous literature we used three independent measures of embodiment (questionnaires, pointing tests and skin conductance response test) to determine if vibrotactile sensory substitution could elicit sense of body ownership of an alien, rubber hand. Our results provided evidence that this is possible and that different modality-mismatched paradigms produce stronger or weaker illusions.

## Methods

### Participants

Twenty volunteers (10 female, 10 male, age = 29±4) naïve to the RHI participated in the study. Nineteen were right hand dominant and one left dominant. All participants were healthy and claimed to have normal hand sensation and vision. Informed consent according to the Declaration of Helsinki (BMJ 1991; 302: 1194) and to the Ethical Committee of the Scuola Superiore Sant’Anna, was obtained before conducting the experiments.

### Vibrotactile Stimulator

**Figure 1:**
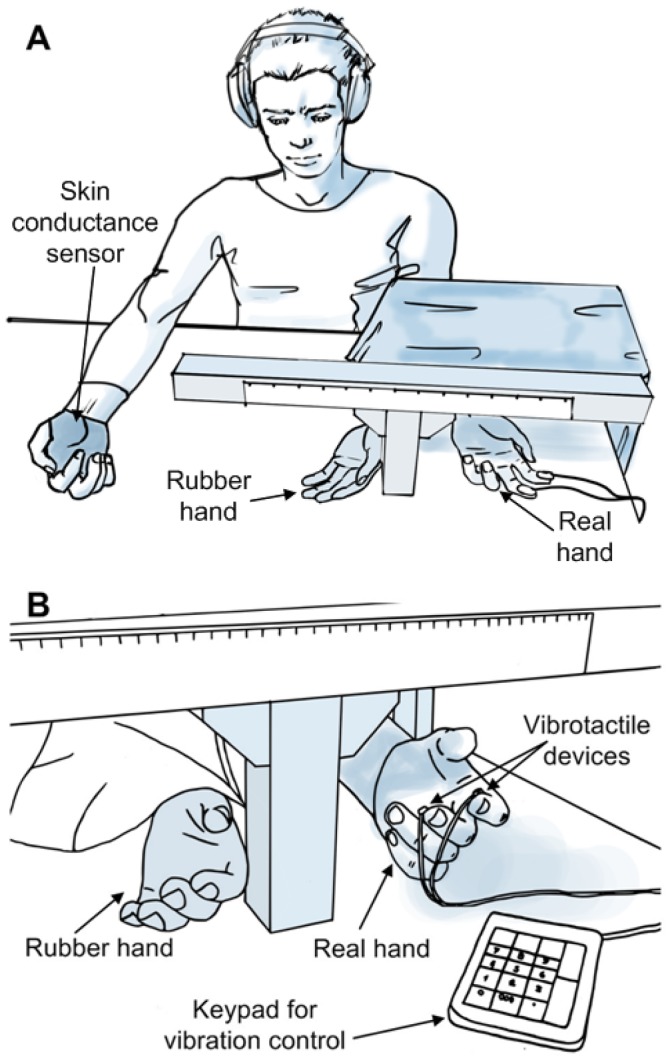
Experimental set-up. Experimental set-up during the modality mismatch experimental condition. A) Participants wore ear-muffs to block out any noise arising from the vibration and a skin conductance sensor on the palm of their right hand. Participants were instructed to fix their sight on the rubber hand, throughout the experiment. B) Close up on the placement of the vibrotactile units on the index and middle fingers of the participant, and on the keypad used to trigger them.

Vibrotactile stimulation was provided by means of a custom built system [10] comprising of two distinct miniature vibrators (8 mm diameter, 3.4 mm height, 0.7 g weight). When used (see procedure below), vibrators were attached with tape on the finger pads of the index and middle fingers (Fig. 1). Each vibrator could be selectively activated to vibrate at a pre-defined vibration frequency (165 Hz) and force amplitude (0.36 N: i.e. largely supra-threshold) or deactivated. Vibrators were triggered off through a keypad: as soon as a key was pressed the corresponding unit would start vibrating. Vibration was maintained as long as the key was kept pressed, and switched-off simultaneously with key release. The time delay between the pressure of a key and the starting of perceivable vibration was negligible (i.e. <10 ms). With this setup the experimenter was allowed with one hand to stimulate the rubber hand in full view, and with the other hand to press keys to induce vibrations on the fingers. A detailed description of the vibrotactile display can be found in [10].

### Skin Conductance Sensor

Armel and Ramachandran [15] showed that participants display a strong skin conductance response (SCR) to a threat stimulus on the rubber hand, when the RHI illusion occurs. Hence we recorded SCR using a device worn on the palm of the hand (Q sensor palm system by Affectiva Inc.). Once activated the sensor sampled data at 32 Hz and stored it inside its internal memory for subsequent off-line analysis. The sensor was worn 5 minutes before the experiments began in order to achieve stable hand-electrode contacts [6].

### Experimental Session: General Procedure

**Figure 2:**
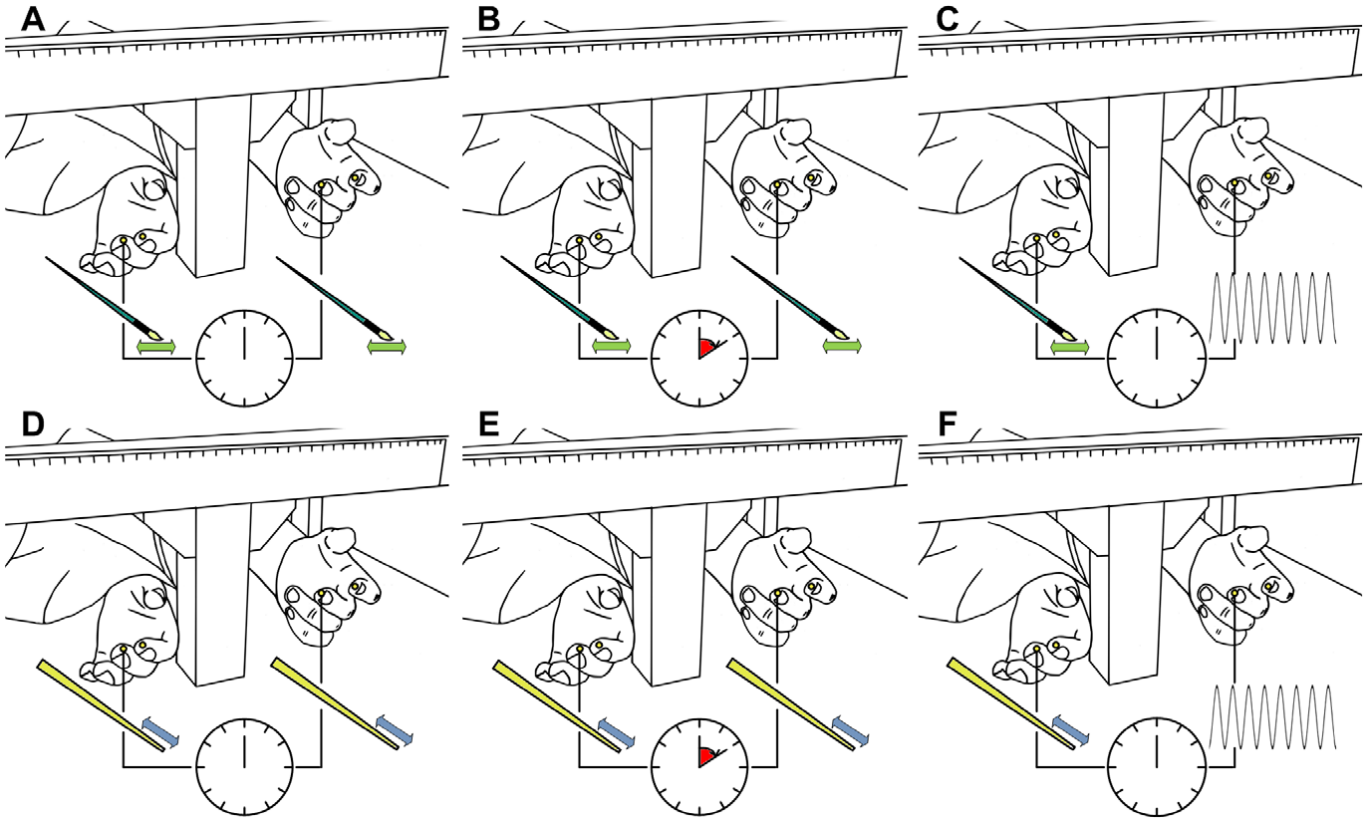
Experimental conditions. Schematic diagrams showing the six experimental conditions used to induce the Rubber Hand Illusion (RHI) in our experiments. A) Synchronous Congruent Brushstroke (SCB) was the classic RHI as designed by Botvinick and Cohen (1998). B) Asynchronous Congruent Brushstroke (ACB). C) Synchronous Incongruent Brushstroke (SIB) was our first experimental (modality mismatched) condition: brush strokes and vibrations were delivered synchronously on the rubber hand and real hand, respectively. D) Synchronous Congruent Tapping (SCT) was a modified version of RHI. E) Asynchronous Congruent Tapping (ACT). F) Synchronous Incongruent Tapping (SIT) was our second experimental condition: taps and vibrations were delivered synchronously on the rubber hand and real hand, respectively.

Each participant sat comfortably on a chair in front of a table, with his/her left arm lying in a supine position behind a screen, ensuring that it was out of sight throughout the entire experiment. The participant wore the skin conductance sensor on the right hand. A life-sized left-handed cosmetic hand prosthesis (i.e. the rubber hand) was placed in front of the participant, approximately 10–20 cm median and parallel to the real arm (Fig. 1A). The participant was instructed to relax and fix his/her sight on the rubber hand during the experiment. Six stimulation conditions (cf. Fig. 2) were tested twice, making a total of twelve stimulation trials. Each trial lasted 45 seconds, and consisted in alternate stimulations of the index and middle fingers of the rubber and the real hand. The twelve trials were randomized within one session (i.e. one subject) and among the participants. Between each trial participants had a 1–2 minute long break to relax, and the overall experimental session lasted around 30 minutes. Participants wore ear-muffs throughout the experiment to block out any noise arising from the vibrators.

The six experimental conditions were *Synchronous Congruent Brushstroke* (SCB), *Asynchronous Congruent Brushstroke* (ACB), *Synchronous Incongruent Brushstroke* (SIB), *Synchronous Congruent Tapping* (SCT), *Asynchronous Congruent Tapping* (ACT) and *Synchronous Incongruent Tapping* (SIT). Congruent conditions used modality matched stimulation; *incongruent* used modality mismatched stimulation (i.e. sensory substitution). Synchronous congruent brushstroke (SCB) condition replicated the original experiment by Botvinick and Coehn [1]: two small paintbrushes were used to stroke the rubber hand and the subject’s hidden hand, simultaneously. The pulps of distal phalanxes of the index and middle fingers were targeted, being the brushstrokes moving proximal to distal. It is known from literature that this condition elicits a strong RHI. It is also known, that self-attribution does not occur if a small asynchrony between brushstrokes is introduced. This assumption was verified with the asynchronous congruent brushstroke (ACB) stimulation i.e. the control condition. The experimental condition - synchronous incongruent brushstroke (SIB)- used modality mismatched stimulation: vibrators were placed on the index and middle finger pads of the subject’s hidden hand (Fig. 1B) and were synchronously activated while brush-stroking the respective finger pads of the rubber hand. In the SCB, ACB and SIB conditions, stimulations were delivered manually by the experimenter at a frequency of about 1 Hz (earphones playing a metronome aided the experimenter) and each brushstroke was about 1 to 2 cm long. The duration of the brushstroke was around 0.6–0.7 seconds matching the duration of the vibration in the experimental condition. We hypothesized that sensory substitution i.e. modality mismatch between visuo-tactile stimuli would elicit the RHI.

In order to infer on self-attribution across different modality-mismatched paradigms, a modified version of the Rubber Hand experiment was introduced and tested within three further conditions (cf. Fig. 2 D–F). The difference was that the brushstrokes on the intermediate and distal phalanxes were substituted by *tapping* with the tip of a chopstick on the finger pads i.e. simpler, shorter, and more localized tactile events, compared to the brushstroke. Tapping on finger pads was chosen as “light touch” resembles frequent daily-life experience during object grasping, manipulation and exploration. In the synchronous congruent tapping (SCT) condition chopsticks were used to tap finger pads of the rubber and the subject’s hand, simultaneously. We hypothesized that this perceptual experience would still elicit the RHI. In the asynchronous congruent tapping (ACT) a small asynchrony between touches was introduced and hence we hypothesized that no illusion would occur. The experimental condition i.e. synchronous incongruent tapping (SIT) used modality mismatched stimulation: vibrators on the index and middle finger pads of subject’s hidden hand were synchronously activated while tapping the respective finger pads of the rubber hand with the chopstick. In all SCT, ACT and SIT conditions taps were delivered manually by the experimenter at a frequency of 1 Hz and the contact duration was about 0.3–0.4 seconds, matching the duration of the vibration (when present). We hypothesized that the modality mismatch between the tap and vibration would still elicit the RHI.

Before each stimulation trial the participants were asked to close their eyes and to indicate with their right hand the felt position of their index finger by means of a *pre-stimulation* pointing task [1]. A ruler mounted on the screen was used to measure the end point of the movement. Immediately after each trial either one, two or none of the following tests of embodiment were carried out: (i) subjective data collection in the form of a questionnaire; (ii) proprioceptive drift by means of a *post-stimulation* pointing task; and (iii) skin conductance response (SCR) test. If two tests were presented then the priority order was the following: SCR, pointing task, questionnaire. This order was chosen as SCR had a duration of around 12 seconds and required the participant to simply keep visual contact of the rubber hand, hence maintaining the illusion for the test following. The three tests were presented/performed in a pseudo-randomized order (also across subjects) in order to collect at the end of the session one measure from each test and each experimental condition, from each individual subject.

### Post-stimulation Questionnaire

Each participant filled-in the questionnaire (one for each stimulation condition soon after the trial) comprising of the nine statements (S1.S9) designed by Botvinick and Cohen [1] in the original experiment, and translated into Italian. The questionnaire required the participants to rate the strength of their agreement or disagreement with nine perceptual effects. Three of the statements (i.e. illusion statements) referred to the extent of sensory transfer into the rubber hand and the self-attribution of it during the trial (S1: It seemed as if I was feeling the touch of the paintbrush/chopstick in the location where I saw the rubber hand touched; S2: It seemed as though the touch I felt was caused by the paintbrush/chopstick touching the rubber hand; S3: I felt as if the rubber hand was my hand). The other six statements (i.e. control statements) served as controls for compliance, suggestibility, and “placebo effect” [S4: It felt as if my (real) hand was drifting towards the right (towards the rubber hand); S5: It seemed as if I might have more than one left hand or arm; S6: It seemed as if the touch I was feeling came from somewhere between my own hand and the rubber hand; S7: It felt as if my (real) hand was turning ‘rubbery’; S8: It appeared (visually) as if the rubber hand was drifting towards the left (towards my hand); S9: The rubber hand began to resemble my own (real) hand, in terms of shape, skin tone, freckles or some other visual feature]. The order in which the nine statements were presented was randomized across trials and participants. Participants were asked to rate the extent to which these statements did or did not apply, using a seven-point analogue scale. On this scale, -3 meant “absolutely certain that it did not apply,” 0 meant “uncertain whether it applied or not,” and +3 meant “absolutely certain that it applied”.

As in Ehrsson et al. study [5], in addition to the nine statements, subjects were asked to rate *vividness* and *prevalence* of self-attribution of the rubber hand. The *vividness* was defined as how life-like and realistic the illusion was when it was experienced; it was rated from 0 to 10. The *prevalence* rating (from 0% to 100%) reflected the percentage of time that the illusion was experienced (equivalent to the continuance of the illusion).

### Post-stimulation Pointing Task and Proprioceptive Drift

After the stimulation trial a new pointing test was performed and the measure of where the index finger was felt was noted. The proprioceptive drift (as defined by [17]) was calculated as the difference between the pre-stimulation and post-stimulation pointing task measurements, and provides behavioural evidence of the occurrence of the illusion. A positive proprioceptive drift represented a mislocalization of the participant’s hand toward the rubber hand, while a negative drift represented a mislocalization of the participant’s hand away from the rubber hand. Post-stimulation pointing tasks were performed after the trial either before the questionnaire, or after the SCR test.

### Post-stimulation Skin Conductance Response Test

This test was included to obtain physiological evidence for the illusion that would be independent of written or pointing task responses [15]. Before the experiments commenced the participants had been informed that they would never be stabbed with a needle and that they would not experience any painful sensation. This test was performed once for each stimulation condition, by each participant (cf. Fig. 2). After the stimulation trial the rubber hand was suddenly stabbed with a needle attached to a syringe (100 ml), just on the palm of the hand. Great care was taken to move the syringe in the same way from trial to trial. The procedure of moving the needle and stabbing the hand, i.e. the *threat stimuli*, lasted for about 2 s; the needle was maintained on the rubber hand for about 5 s before being removed from sight of the subject. For each trial we identified a peak value in the SCR within 1–10 s of the onset of the threat stimuli. As a baseline we used the value 1 s before the threat stimuli was presented (following the protocol designed by [6]). The magnitude of the SCR was used to measure the extent of the illusion [15].

## Results

### A. Questionnaire

**Figure 3:**
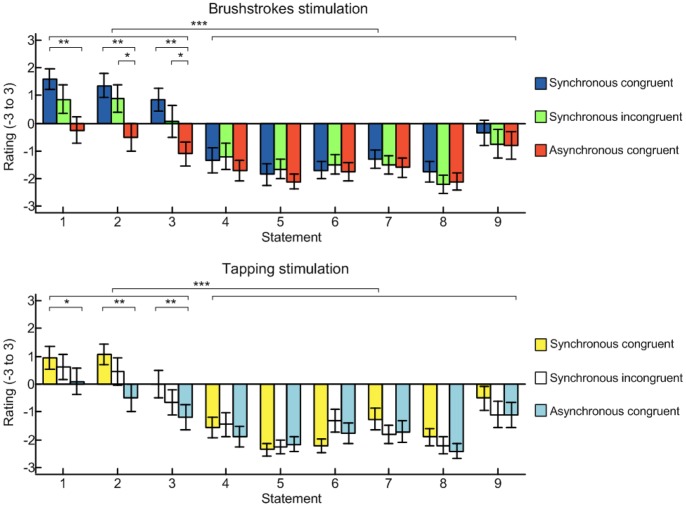
Results of questionnaire for different conditions. Results of the questionnaire in the different stimulation conditions. By comparing the upper and lower panel it can be noted that the tapping conditions were overall lower rated lower than the brushstrokes conditions. Asterisks legend: * indicates p<0.05; ** indicates p<0.01; *** indicates p<0.001.

The graphs in Fig. 3 present the mean ratings (± standard error) from the questionnaire after the brushstroke conditions (upper panel) and the tapping conditions (lower panel). We used planned comparisons to compare the illusion statements across conditions and to compare the illusion statements against the control statements. The a priori hypotheses were that illusion statements would be rated higher than the control statements, and that the ratings from the illusion statements would be greater in synchronous than in the asynchronous conditions. In agreement with previous studies [1], [5], [11], [17], [21], after the classical RHI condition (SCB) participants provided stronger ratings for the three illusion statements than for the six control statements (Wilcoxon signed rank test; p<0.001), hence indicating that they were not suggestible; in addition the ratings from the illusion statements were greater in the SCB (blue bars) than in the ACB condition (red bars; p<0.01 Wilcoxon signed rank test comparing each of statements 1–3 for the SCB and ACB). The questionnaire outcomes confirmed the hypothesis of this study for the SIB condition: after incongruent stimulation, subjects rated the illusion statements greater than the control statements (p<0.001; Wilcoxon signed rank test) and most important, the illusion statement ratings (green bars) were greater than in the ACB control condition (red bars; p<0.05 Wilcoxon signed rank test comparing subject’s mean statements 1–3 for the SCB and ACB). Wilcoxon rank test revealed that the scores were significantly different for statement 2 and 3 (p<0.01) and close to the significant level for statement 1 (p = 0.059). Subjective judgments through the questionnaire showed that visuo-tactile modality mismatch wherein felt vibrations were synchronously delivered with seen brushstrokes can promote self-attribution of an alien hand.

The trials in which the modified Rubber Hand paradigm was employed i.e. when chopsticks were used to tap the finger pads, confirmed that the RHI is promoted also when a more localized and short stimulation is delivered synchronously to the rubber and to the real hand (cf. Fig. 3 lower panel). Indeed during the SCT condition participants showed that they were not suggestible (rating of the illusion statements greater than control statements; Wilcoxon signed rank test; p<0.001) and as for the classic Rubber Hand experiment the ratings from the illusion statements were greater in the SCT (yellow bars) than in the ACT condition (pale blue bars; Wilcoxon signed test: comparing statement 1 - p<0.05, statement 2 and 3– p<0.01). Outcomes from the questionnaire deny the study hypothesis for the SIT condition: after incongruent modality, subjects rated the illusion statements greater than the control statements (p<0.001; Wilcoxon signed rank test), and although the illusion statement ratings (white bars) had a mean value greater than in the control condition ACT (pale blue bars), these differences were not statistically relevant (Wilcoxon signed rank test: comparing statement 1– p = 0.418, statement 2– p = 0.075, and 3– p = 0.187).

**Figure 4:**
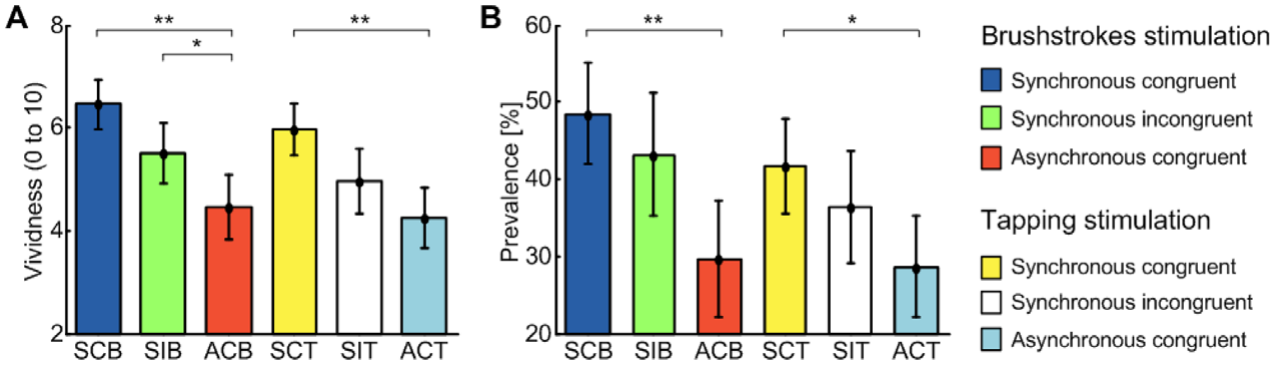
Results of prevalence and vividness for different conditions. Mean ratings of vividness (A) and prevalence (B) to the six stimulation conditions by the participants (N = 20). Asterisks legend: * indicates p<0.05; ** indicates p<0.01.

The histograms in Fig. 4 show the subjects’ (N = 20) mean rating to the vividness (left panel) and prevalence (right panel) of the illusion, in the different conditions. For the vividness the lowest value, was obtained for ACT (4.2±0.6; mean ± standard error), the highest one: 6.4±0.5 (SE), for SCB. Wilcoxon signed rank test demonstrated statistically significant difference between SCB and ACB (p<0.01), between SIB and ACB (p<0.05), and between ACT and SCT (p<0.01), but no significant differences between SIT and ACT (p = 0.294 Wilcoxon signed rank test). Regarding the prevalence scores: ACT received the lowest score (28±7%), SCB the highest one (48±7%). Statistically relevant differences were found between SCB and ACB (p<0.01) and between SCT and ACT (p<0.05) but not between SIB and ACB (p  = 0.069 Wilcoxon signed rank test) and between SIT and ACT (p = 0.260 Wilcoxon signed rank test).

### Post-stimulation Pointing Task and Proprioceptive Drift

**Figure 5:**
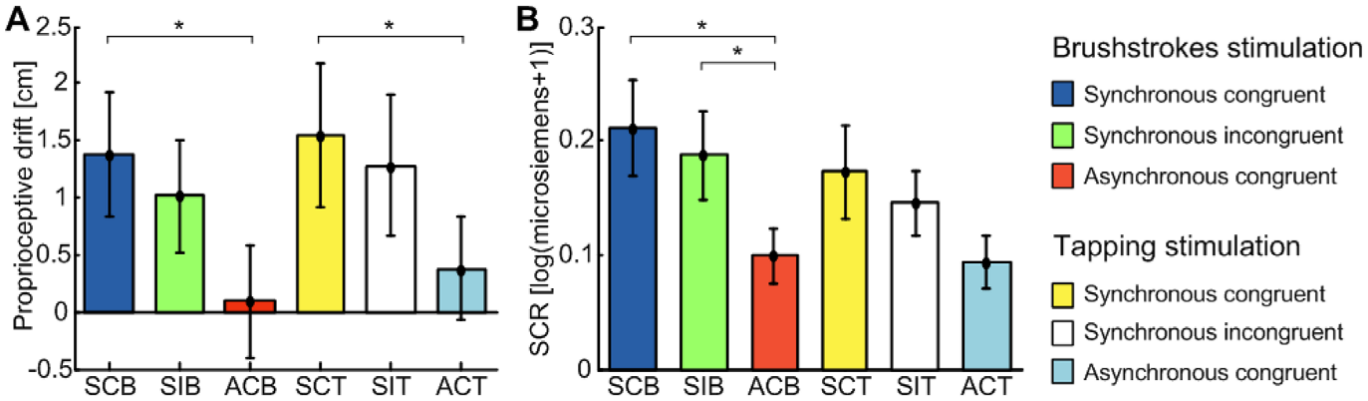
Results of proprioceptive drift (left) and of psychological induced sweating (right) for different conditions. Left panel. Mean proprioceptive drift (in cm) measured as a difference between the pre-stimulation and post-stimulation pointing tasks. The histogram shows data from all subjects in the different experimental conditions (* indicates p<0.05). **Right panel.** Mean psychologically induced sweating, as measured by the skin conductance response (SCR in microsiemens) after the threat stimuli on the rubber hand on all participants (N = 20), in the six different experimental conditions (* indicates p<0.05).

In agreement with literature, greater proprioceptive drifts were achieved with synchronous than with asynchronous stimulation (Fig. 5 - left panel). The difference between the synchronous and asynchronous condition was 1.3±0.7 cm for the brushstroke stimulus and 1.2±0.5 cm for the tapping stimulus. These differences were statistically significant (paired 2-tailed t-test comparing SCB with ACB, and SCT with ACT p<0.05). Both incongruent conditions seemed to result in proprioceptive drifts greater than their respective asynchronous control conditions (mean difference: 0.9±0.4 for the brushstroke stimulation and 0.9±0.8 for the chopstick stimulation). These differences, however, were not statistically significant in either case, although the difference between SIB and ACB was close to the significance level (p = 0.07 paired t-test).

### Post-stimulation Skin Conductance Response Test

Fig. 5 (right panel) shows the mean SCR [log(microsiemens+1) as in [15]] of all subjects resulted from threatening the rubber hand in the different conditions. With the brushstroke stimulation a larger SCR was measured after both the congruent and incongruent synchronous conditions compared to the asynchronous condition. This difference was statistically significant (paired 2-tailed t-test: p<0.05) as found in previous studies, and coherent with the other two tests. As hypothesized, a significant difference was found also between SIB and ACB (p<0.05). With regards to the tapping stimulation, a similar trend was found across conditions although with no significant differences.

### Results Across the Three Measures

The data from the three experiments were finally combined for analysis using a 2 way ANOVA [factors: stimulation conditions (SCB, SIB, ACB, SCT, SIT and ACT) and the three measurements (questionnaires, proprioceptive drift, SCR)] as suggested by the work by Ehrsson et al. [6]. The mean values from the three illusion statements were used as a measure from the questionnaires. From the pointing task and SCR tests we took the proprioceptive drift and the SCR value from each condition and individual subject. Statistically significant differences were found between the six conditions [F(5,342)  = 3.68, p = 0.003] and the three measurements [F(2,342) = 7.06, p = 0.001; the interaction between factors was not statistically significant F(10,342) = 0.96, p = 0.47]. Hence, a post-hoc Tukey’s least significant difference procedure was applied. This showed that the measures were significantly different between SCB and ACB (p<0.001), SIB and ACB (p<0.05) but not between SCB and SIB (p>0.05) in the brushstroke conditions. This demonstrates that as assessed across all measures, the synchronous modality matched and modality mismatched paradigms elicited similar levels of illusion. In other words, vibrotactile sensory substitution can elicit feeling of ownership of an alien hand. The post-hoc procedure on the tapping conditions showed differences only between the synchronous and asynchronous congruent modalities (SCT vs. ACT p<0.01). We cannot therefore derive that the synchronous incongruent tapping condition elicited a significant illusion. Finally, the post hoc procedure highlighted statistical differences between ACB and SCT (p<0.01) between ACB and SIT (p<0.05) and between ACT and SCB (p<0.05). The other comparisons between conditions did not yield to statistically different levels of illusion.

## Discussion

Taken collectively our results provide evidence that vibrotactile sensory substitution can be used to induce self-attribution of a rubber hand when synchronous but modality conflicting visuo-tactile stimulation is delivered to the biological finger pads and to the equivalent rubber hand phalanges. In particular we demonstrated that after just a short exposure (45 seconds) to modality-mismatch stimulation using seen brushstrokes and felt vibrations, participants felt a vivid RHI. This is an important finding for the field of prosthetics; in fact arrays of vibrotactile stimulators could be easily incorporated into upcoming artificial hands with tactile sensors [10], in order to stimulate referred phantom fingers, so to induce the RHI on a daily usage, as elegantly proposed by Ehrsson and colleagues [6]. With a modified version of the RHI in which we exposed participants to a different daily-life-like visuo-tactile stimulation, we showed that synchronous *tapping* of fingers also induced a vivid illusion. However the illusion seemed less vivid compared to the traditional RHI. This seems reasonable considering that a somatotopic matching in stimulation parameters like direction and path of brush stroking represent a richer perceptual experience compared to the condition in which the finger is simply touched on just a single point. The neural encoding of such stimuli (as argued below) is also a possible explanation. Results from the modified paradigm underlined that the vividness of the illusion promoted by modality-mismatched stimulation is actually correlated to the conflict between visual and tactile stimuli. When tapping was delivered synchronously with vibration cues a weaker RHI was elicited, compared to the brushstroke-vibration condition.

Outcomes were based on three independent measures of embodiment that resulted in closely matching results; this both represents a methodological strength for this study and corroborates the robustness of our results. With the questionnaires participants provided a self evaluation of the illusion by rating perceptual statements and the control statements allowed to confirm they were not suggestible. The behavioural mislocalization of the proper hand towards the rubber hand was evaluated through the pointing task. Finally the SCR provided an objective physiological evidence of autonomic system arousal [15]. Another methodological asset of the present study is that the twelve stimulation trials representing six different experimental conditions (each condition was presented twice to the subject) and the measure tests were delivered in a randomized manner across subjects so that our results are virtually free from any learning effect.

The traditional RHI experiment served as a baseline for the other condition; the results relative to the questionnaires confirmed the non-suggestibility of our subjects (hence the validity of the data) as shown by the significant difference in ratings between the illusion and control statements. Rates to vividness and prevalence across the different conditions (SCB, SIB and ACB) produced results consistent to the ratings of the illusion statements. Interesting is that the illusion in the traditional experiment measured by the questionnaires but also by the proprioceptive drift and the SCR was weaker compared to previous studies [6],[13]; we suggest that this is possibly due to the shorter duration of our trials compared to previous works (45 s vs. 60–600 s) [15], [17]. Tsakiris and Haggard [17] showed that the proprioceptive drift towards the rubber hand increases with stimulation time, indeed. Another explanation of such weaker illusion could be due to the posture of the hand that (unlike most of the previous studies) was palm-up and hence stimulated on the finger pulps; in fact the sensibility of the fingertip is significantly higher compared to the dorsal areas of the fingers and it might be easier for the subject to note possible inconsistencies between the visuo-tactile stimulation (eventually, the differences of our setup -hand posture and duration of the trial- might have caused the lack of significant results in some of the measures). Nevertheless, it must be underlined that both the SCR and the proprioceptive drift after synchronous stimulation were also significantly greater than the asynchronous conditions.

An important point is that modality mismatched stimulation (SIB) across all measures of embodiment was statistically greater than the asynchronous condition (ACB). This result corroborates Armel and Ramachandran [17] findings which hypothesized that the illusion arises mainly from the “Bayesian logic” of perception: the brain’s remarkable ability to detect statistical correlations in sensory inputs in constructing useful perceptual representations of the world–including one’s body. Indeed, this bottom-up process is resistant to top-down knowledge of body reactions to stimuli and to the irrationality of the situation: a gentle paintbrush brushstroke does not produce a mechanical vibration like the one we provided! Our study also integrates and extend the results by Schutz-Bosbach and colleagues [22]; they manipulated the congruency of the observed and felt tactile stimulation by stroking the fake and real hand using fabrics with different roughness and showed that incongruencies between the visual and tactile stimulation did not affect the RHI. Similar principles are thought to apply to audiovisual ventriloquism [23] wherein auditory events are mislocalized toward their apparent visual source only when the location of the visual event falls within the possible range of the sound location. Nevertheless in agreement with a top-down model [17] the synchronous mismatched tapping did not statistically differ from the asynchronous tapping. These dissimilarities (different levels of illusion with different synchronous paradigms) suggest that synchronicity is necessary but is not a sufficient condition to induce a strong RHI; the vividness of the illusion seem to be modulated by the specificity of visuo-tactile conflict and on how the matching of the concurrent inputs is perceived as realistic based on a pre-existing representation of one’s own body.

We suggest that the lower rate of illusion induced by synchronous tapping compared to brushstroke was related to the different mechanically spatio-temporal characteristics of the stimuli and consequently to the richness of information (over time) encoded by the sensory organs (mechanoreceptors) in the finger pads, which were eventually used to recalibrate the body map. The tapping stimulus, is a relatively quick, weak pointed touch, that provokes skin deformations and present static spatial characteristics. This tapping similar to the task of grasping an object is mainly mediated by FA-I and SA-I afferents, through quick firing bursts when the contact is made and broken [24]-[26]. Conversely gentle brush strokes onto a finger pad (namely movements across a receptive field) are spatially dynamic stimuli that evoke afferents along the path of motion, mainly FA, in a substantially continuous manner as the brush moves until the movement is stopped. It was shown by Essick and Edin [27] that the evoked firing rate is proportional to the speed of the brush stroke. The spatiotemporal distributed information is perceptually important as suggested by the deficits in direction discrimination observed in patients with peripheral nerve injuries after regeneration [28]. Hence intrinsically a brush stroke evokes richer spatiotemporal patterns compared to the tapping. Moreover, it should be considered that in our setup the duration of which brush or chopstick were in contact with the skin was very different: the brushstroke lasted more than twice as long as the tapping (around 0.6–0.7 vs. 0.3–0.4 seconds respectively) whereas the stimulation frequency was 1 Hz in both cases.

Another hypothesis for the differences in vividness between mismatched brush stroke and mismatched tapping might be related to the specificity of mechanoreceptors to tactile stimuli. Indeed FA-II mechanoreceptors in the glabrous skin (Pacini endings) are extremely sensitive to high-frequency vibrations (50–400 Hz) propagating through the skin [24], [26], and (together with FA-I) are known to respond to brush strokes [27]. On the other hand they are barely sensitive to dynamic skin deformations of relatively high frequencies (5–50 Hz) like those evoked by weak pointed touch, as in our tapping conditions (FA-I mediate these stimuli). As our vibrations fell exactly in the FA-II range of responsiveness we hypothesize that brushstrokes elicited a more vivid RHI than tapping, because brushstrokes and vibrations elicited the same sensory organs (FA-II) that in turn mediated the encoded version of the stimuli to the central nervous system. Future studies employing lower vibration frequencies will be carried out to assess this argument.

It is worth mentioning that the short duration of the stimulation trials, probably played a significant role in emphasizing the differences in vividness across conditions. By observing the measures of embodiment one could hypothesize that longer durations would induce significant illusion also in the synchronous incongruent tapping condition. The duration was selected in order to keep the length of all the experiment (12 trials) within 30 minutes, so as to avoid loss of attention in our subjects. Future studies will investigate the effects of trial duration on the vividness of the SIT condition, pursuing methods similar to the study by Tsakiris and Haggard [17].

Many transradial amputees experience tactile phantom sensations when their residual limb is touched. Imaging studies by Vilayanur Ramachandran showed that these sensations are due to rearrangement of cortical circuits occurring in the first hours after amputation. Ramachandran has called this *remapping of referred sensations* (also known as *referred phantom sensations*) [29]. In these cases by transferring the information from sensors in the fingers of a prosthetic hand to specific locations on the skin, fingers of the phantom hand can be stimulated, as suggested by Ehrsson et al., [6]. The approach is exceptionally promising: although it relies on non-invasive technology and stimulation techniques, it would allow for rudimentary recovery of “reorganized” physiological channels. While we were able to induce in healthy individuals self-attribution of a rubber hand using a modality mismatched paradigm, similar studies with amputees experiencing referred phantom finger sensations [6], [29] will be required to determine if the illusion still applies. The present study together with other works in the cross-disciplinary field suggest that this is possible, and hence that the objective of establishing a sense of embodiment for a prosthetic hand using practical devices is achievable.
